# Noncoding RNAs and the Architecture of Gene Regulation: Focus on Long and Small Regulatory RNAs

**DOI:** 10.1002/wrna.70051

**Published:** 2026-07-15

**Authors:** Samuel Z. Desind, Samira K. Bell, Carol S. Lutz

**Affiliations:** ^1^ Department of Microbiology, Biochemistry, and Molecular Genetics, Rutgers Biomedical & Health Sciences New Jersey Medical School, School of Graduate Studies Newark New Jersey USA

## Abstract

Noncoding RNAs (ncRNAs) are a diverse and abundant class of molecules that play essential roles in gene regulation across eukaryotic organisms, including roles in human homeostasis and disease. Historically regarded as transcriptional artifacts or byproducts, ncRNAs are now recognized as essential regulators of transcriptional and post‐transcriptional processes that shape cellular homeostasis and disease phenotypes. We outline the historical discovery, classification, and functional diversification of many classes of ncRNAs, with a focus on long noncoding RNAs (lncRNAs) and microRNAs (miRNAs). We summarize the current understanding of miRNA and lncRNA biogenesis, processing, and key mechanisms of action, including miRNA‐lncRNA interactions and structure‐dependent regulatory functions of lncRNAs. Emphasis is placed on the dynamic interplay between lncRNAs and miRNAs, their roles in fine‐tuning gene expression networks, and how advances in transcriptomics, chemical probing, and structural biology have transformed our understanding of ncRNA regulatory complexity. Finally, we summarize the current landscape of RNA therapeutics. This analysis maps the historical evolution of ncRNA biology, the establishment of ncRNAs as integral regulators of gene expression and disease phenotype, and the emerging prevalence of RNA‐based therapeutic strategies.

## Graphical abstract

NcRNAs play fundamental roles in many facets of cellular homeostasis, systemic physiology and disease outcomes. Historical and current advancements have led to a deeper understanding of ncRNAs' roles, utilizing their diverse and tissue‐specific functions to drive the development of innovative therapeutic tools.

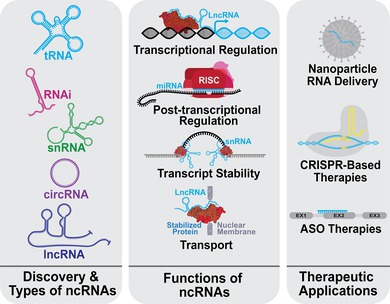

## 1 Introduction

Noncoding RNAs (ncRNAs) are a largely understudied group of biomolecules essential for cellular homeostasis. They have a variety of roles in normal cellular functions and in the onset and progression of many disorders and diseases. Investigating the roles and mechanisms of ncRNAs can be challenging, as these molecules are often low‐abundance, tissue‐specific, and dependent on cellular conditions and microenvironments. These molecules are often transient and influence cellular and physiological functions in fundamental ways that have historically been attributed solely to the functions of other, more abundant biomolecules, such as proteins.

Advancements in polymerase chain reaction (PCR) techniques and continuously evolving sequencing strategies have significantly enhanced our ability to identify and characterize splice variants, post‐translational modifications, structural motifs, and ultra‐long and low‐abundance ncRNA transcripts. Additionally, chemical probing techniques to quantify and visualize the structures of longer RNAs, such as selective 2′‐hydroxyl acylation analyzed by primer extension and mutational profiling (SHAPE‐MaP), have made RNA motif characterization accessible and reproducible. However, these advances are the accumulation of decades of research and innovation that progressively expanded our understanding of ncRNA diversity and functional scope.

## 2 History & Classification of Noncoding RNAs

### 2.1 Early Functional ncRNAs: Translation, Splicing, and Catalytic RNA

The first functionally characterized RNAs were transfer RNA (tRNA) and ribosomal RNA (rRNA), which facilitated the transfer of genetic information from DNA to protein. The abundance of RNA and its localization to the cytoplasm were first observed in the late 1930s and early 1940s (Brachet 1941; Caspersson and Schultz 1938). Evidence of RNA involvement in protein synthesis accumulated throughout the 1940s. Studies quantitatively correlating changes in cellular RNA content with rates of protein synthesis linked nucleic acid metabolism and protein biosynthesis for the first time in the late 1940s (Caspersson 1947). However, the fundamental roles of messenger RNA (mRNA) and ncRNAs, such as tRNA and rRNA, in translation were progressively defined through experimental and theoretical advances in the late 1950s and early 1960s (Hoagland et al. 1958; Watson 1963).

Following Francis Crick's theoretical prediction of a small adaptor molecule linking nucleic‐acid sequence information to amino acids during protein synthesis (Crick 1958), Hoagland et al. demonstrated that amino acids are first activated in an ATP‐dependent reaction, then covalently attached to a discrete, soluble RNA fraction, which are tRNAs. These aminoacyl‐tRNAs, formed by highly specific aminoacyl‐tRNA synthetases, act as intermediate carriers to the ribosome. This ribonucleoprotein complex catalyzes peptide bond formation and was first known as the ribonucleoprotein particle or microsome due to its presence in the microsomal fraction in early cell fractionation studies (Claude 1943). This discovery was followed by the landmark identification of mRNA as the unstable intermediate between DNA and microsomes (Brenner et al. 1961), demonstrating a rapidly synthesized RNA species with a short half‐life distinct from ribosomal RNA. These findings enabled the first experimental assignments of codons within the genetic code (Nirenberg and Matthaei 1961), which relied on synthetic RNAs produced using enzymatic methods developed by Grunberg‐Manago et al. to demonstrate how specific RNA codons specify individual amino acids (Grunberg‐Manago et al. 1956). Subsequently, Watson integrated ribosome sedimentation and cell‐free translation experiments to synthesize and popularize existing biochemical evidence supporting the ribosome as the site where tRNA‐bound amino acids are assembled into polypeptide chains according to the genetic template (Watson 1963).

Researchers identified another class of ncRNAs, small nuclear RNAs (snRNAs), in the 1960s (Clason and Burdon 1969). Subsequent work demonstrated that snRNAs function as integral components of small nuclear ribonucleoproteins (snRNPs) that recognize splice sites through direct base‐pairing with conserved sequences at intron‐exon junctions, thereby mediating intron removal and exon ligation during pre‐mRNA processing through the spliceosome (Lerner and Steitz 1979; Rogers and Wall 1980). This work established snRNAs as active regulators of eukaryotic gene expression rather than structural ncRNAs.

Other significant developments between the early 1980s and early 1990s expanded the known functional diversity of ncRNAs. The discovery of ribozymes as catalytic molecules demonstrated that RNA could possess intrinsic catalytic activity independent of proteins, altering the widespread view of RNA as solely an informational or structural molecule (Cech and Steitz 2014; Kruger et al. 1982). In parallel, small nucleolar RNAs (snoRNAs) were shown to be required for pre‐rRNA cleavage and processing during ribosome biogenesis (Tollervey 1987), and later studies established that many snoRNAs act as guide RNAs that direct site‐specific chemical modifications, including 2′‐O‐methylation, of rRNAs (Kiss‐László et al. 1996). Additionally, antisense RNAs were identified as regulators of gene expression through sequence‐specific RNA–RNA interactions that influence transcript stability and post‐transcriptional control in mammalian systems (Munroe and Lazar 1991).

### 2.2 Small RNAs as Regulators: Micro RNAs, Piwi‐Interacting RNAs & RNA Interference

The beginning of the 1990s marked the discovery of small regulatory RNAs and their sequence‐specific interactions with mRNA. Early studies of the gene lin‐14 in *C. elegans* showed that deletion of 3′ untranslated regions from lin‐14 mRNAs resulted in aberrant LIN‐14 protein expression. This deletion removed a negative regulatory element required for normal LIN‐14 temporal decay. Because lin‐4 mutations are known to alter LIN‐14 protein accumulation, lin‐4 was proposed as a regulator of lin‐14 through these 3′ UTR regulatory elements (Ruvkun et al. 1991). This model was further investigated in the laboratory of Victor Ambros, Lee and colleagues who identified that the 21‐nucleotide (nt) lin‐4 encodes a small ncRNA with sequence complementarity to elements in the lin‐14 3′ UTR. They demonstrated that it downregulates lin‐14 expression through sequence‐specific base pairing with complementary elements in the 3′ untranslated region (UTR) of lin‐14 mRNA, resulting in translational repression without altering mRNA abundance, providing the first direct evidence that small ncRNAs regulate gene expression (Lee et al. 1993). For several years, lin‐4 was considered a developmentally regulated “small temporal RNA” (stRNA) unique to nematodes. The subsequent identification of similar small RNAs across species led to their collective classification as microRNAs in 2001 (Lagos‐Quintana et al. 2001; Lau et al. 2001; Lee and Ambros 2001).

The solidification of miRNAs as a distinct class of ncRNAs catalyzed broader searches for other small RNA‐mediated regulators of gene expression, ultimately defining a larger network of RNA regulatory machinery, the RNA interference (RNAi) pathways. RNAi encompasses small RNAs that direct sequence‐specific repression of target transcripts through Argonaute proteins and shared effector machinery. In canonical RNAi pathways, double‐stranded RNA (dsRNA) is processed by Dicer into small interfering RNAs (siRNAs), which typically guide site‐specific endonucleolytic cleavage of perfectly matched mRNAs. In contrast, microRNAs (miRNAs) typically function through imperfect base pairing to repress translation and accelerate deadenylation (Behm‐Ansmant et al. 2006; Huntzinger and Izaurralde 2011; Stevens 2001).

Novel work by Fire and Mello demonstrated that dsRNA acts as an exceptionally potent trigger of the RNAi pathway. Injection of dsRNA targeting the unc‐22 gene into adult *C. elegans* resulted in robust and specific silencing of gene expression, whereas single‐stranded sense or antisense RNAs were less effective (Fire et al. 1998). This work established dsRNA as the functional initiator of RNAi and earned Fire and Mello the 2006 Nobel Prize in Physiology or Medicine.

In 2006, a distinct class of approximately 24–31 nt, small ncRNAs, piwi‐interacting RNAs (piRNAs), was discovered. PiRNAs associate specifically with Piwi‐clade Argonaute proteins in the germline and are produced independently of Dicer, distinguishing them from miRNA biogenesis pathways. In 2007, piRNAs were shown to function primarily in transposon silencing and germline genome defense, establishing them as a dedicated small‐RNA pathway necessary for supporting genomic integrity (Aravin et al. 2006; Brennecke et al. 2007; Girard et al. 2006; Grivna et al. 2006).

### 2.3 Transcriptome‐Wide Discovery of LncRNAs & CircRNAs

Although many studies focused on the regulatory potential of small RNAs, other efforts began to interrogate the broader landscape of noncoding transcription. Early genome‐wide analyses primarily focused on detecting and characterizing protein‐coding genes, which constitute only a small fraction of the genome, leaving much of the transcriptome unexplored. Advances in transcriptomic sequencing enabled researchers to detect widespread transcription outside annotated protein‐coding regions. Studies by Kapranov and colleagues and by the FANTOM Consortium demonstrated the abundance of previously unannotated, longer noncoding RNA transcripts in both the human and mouse transcriptomes (Carninci et al. 2005; Kapranov et al. 2002). These findings challenged the prevailing “junk DNA” paradigm by revealing widespread transcription outside protein‐coding regions. Kapranov and colleagues later referred to these transcripts as “long RNAs” (lRNAs), defining a population of noncoding RNAs exceeding 200 nucleotides in length. Together with related early transcriptomic studies, this work helped establish a distinct class of regulatory transcripts now recognized as long noncoding RNAs (lncRNAs).

Although the prevalence of lncRNAs in the transcriptome was well established by the late 2000s, the apparent lack of evolutionary conservation among many lncRNA transcripts led to the assumption that they were not functionally relevant and not biologically significant. By the late 2000s, only a handful of lncRNAs had been well‐characterized and were known to have functional roles. Despite prevailing skepticism about the functional relevance of noncoding transcription, Guttman and colleagues used large‐scale chromatin‐state mapping in the late 2000s to identify over 1000 long noncoding RNAs (Guttman et al. 2009). They demonstrated that lncRNA expression is tissue‐specific and linked to multiple biological processes, including stem cell pluripotency, immune regulation, and neuronal function. Their analysis showed that lncRNAs are regulated by core transcription factors, including p53, NFκB, Sox2, and Oct4, highlighting their integration into transcriptional networks governing cellular homeostasis. Their work identified and provided functional evidence for over 150 lncRNAs (Guttman et al. 2009).

Another critical study from the ENCODE Project, led by Djebali and colleagues, revealed that approximately 75% of the human genome is transcribed. They identified thousands of previously unannotated lncRNAs with tissue‐specific expression primarily localized in the nucleus (Djebali et al. 2012). This work also identified enhancer RNAs (eRNAs) transcribed from active regulatory regions, linking ncRNA transcription to chromatin state and regulatory machinery. Large‐scale transcriptomic analyses, including studies by Guttman and colleagues, redefined the concept of a gene by highlighting the widespread functional potential of noncoding transcripts and provided significant evidence for the extensive examination of lncRNAs' roles in gene regulation, human health, and disease (Djebali et al. 2012; Guttman et al. 2009; Jarroux et al. 2017).

In the early 2010s, large‐scale transcriptomic studies revealed another previously overlooked and abundant class of noncoding RNAs: circular RNAs (circRNAs) (Jeck et al. 2013; Salzman et al. 2012; Yang et al. 2022). Although circRNAs were first observed in the late 1970s and early 1980s, their widespread expression, roles in human gene regulation, health, and disease have only been elucidated over the past decade (Hsu and Coca‐Prados 1979; Kristensen et al. 2019; Memczak et al. 2013; Wilusz 2015). Initially considered uncommon byproducts of RNA splicing and exon scrambling (Nigro et al. 1991), circRNAs were later shown to exhibit tissue‐specific expression and to regulate gene expression through interactions with miRNAs (miRNA sponging) and RNA‐binding proteins, as well as through interactions with transcription factors (Guo et al. 2014; Memczak et al. 2013). Due to their covalently closed structure, circRNAs are an unusually stable class of ncRNAs, adding a layer of regulatory complexity to the noncoding transcriptome (Hansen et al. 2013; Kristensen et al. 2019; Li et al. 2015).

**FIGURE 1:**
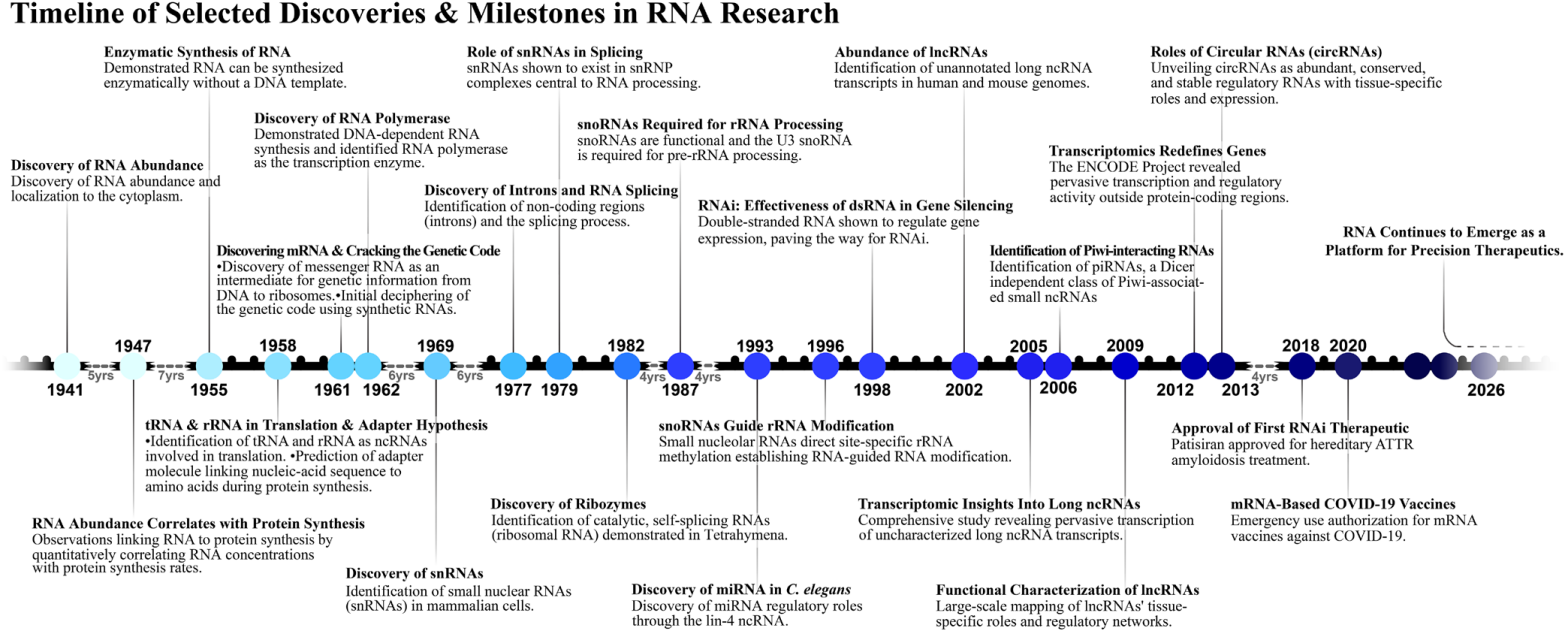
Timeline of RNA Discoveries & Milestones. This timeline highlights discoveries and milestones mentioned in the text, as well as additional events important to the history of ncRNA. The gray dashed lines indicate an axis break; the duration of the break is listed below the dashed line. References corresponding to timeline events are listed in Table 1. Abbreviations: NcRNA, noncoding RNA; tRNA, transfer RNA; rRNA, ribosomal RNA; mRNA, messenger RNA; snRNA, small nuclear RNA; snRNP, small nuclear ribonucleoprotein; snoRNA, small nucleolar RNA; dsRNA, double‐stranded RNA; RNAi, RNA interference; miRNA, microRNA; piRNA, Piwi‐interacting RNA; circRNA, circular RNA; ENCODE, Encyclopedia of DNA Elements; ATTR, amyloid transthyretin.

Decades of discovery have reframed RNA from an ephemeral messenger into a central component and vast network of gene regulatory systems. These findings collectively demonstrate that noncoding RNAs are not peripheral byproducts of gene expression, but fundamental components that shape transcriptional programs, cellular specificity, and physiological homeostasis. lncRNAs remain the most diverse and least understood class of RNAs, with a wide range of proposed and still unresolved biological functions. For these reasons, the following section will focus specifically on lncRNAs and how lncRNA structure governs gene regulation. A timeline of key milestones discussed in this section is shown in Figure 1 and summarized in Table 1.

**TABLE 1:** Summary of Discoveries and Milestones.

Year	Discoveries or events	Reference, PubMed ID
1941	Discovery of RNA Abundance	Brachet (1941), n.d. and Caspersson and Schultz (1938), n.d.
1947	RNA Abundance Correlates with Protein Synthesis	Caspersson (1947), 20,257,022
1956	Enzymatic Synthesis of RNA	Grunberg‐Manago et al. (1956), 13,315,374
1958	Adaptor Hypothesis Proposed	Crick (1958), 13,580,867
1958	Roles of tRNA and rRNA in Translation	Hoagland et al. (1958), 13,538,965 and Watson (1963), 13,999,211
1961	Identification of mRNA	Brenner et al. (1961), 20,446,365
1961	Initial Deciphering of the Genetic Code	Nirenberg and Matthaei (1961), 14,479,932
1962	Discovery of RNA Polymerase	Hurwitz et al. (1962), 13,955,883
1969	Discovery of snRNAs	Clason and Burdon (1969), 5,811,903
1977	Discovery of Introns and RNA Splicing	Chow et al. (1977), 890,740
1979	Role of snRNAs in Splicing	Lerner and Steitz (1979), 316,537 and Rogers and Wall (1980), 6246511
1982	Discovery of Ribozymes	Kruger et al. (1982), 6,297,745
1987	snoRNAs Required for rRNA Processing	Tollervey (1987), 3,327,689
1993	Discovery of miRNA in *C. elegans*	Lee et al. (1993), 8,252,621
1996	snoRNAs guide rRNA modification	Kiss‐László et al. (1996), 8,674,114
1998	RNAi: Effectiveness of dsRNA in Gene Silencing	Fire et al. (1998), 9,486,653
2002	Abundance of lncRNAs	Okazaki et al. (2002), 12,466,851
2005	Transcriptomic Insights Into lncRNAs	Carninci et al. (2005), 16,141,072
2006	Identification of Piwi‐interacting RNAs	Aravin et al. (2006), 16,751,777
2009	Functional Characterization of lncRNAs	Guttman et al. (2009), 19,182,780
2012	Transcriptomics Redefines Genes	Consortium (2012), 22,955,616
2013	Roles of Circular RNAs (circRNAs)	Memczak et al. (2013), 23,446,348
2018	Approval of First RNAi Therapeutic	Adams et al. (2018), 29,972,753
2020	mRNA‐Based COVID‐19 Vaccines	Polack et al. (2020), 33,301,246
2026	RNA Emerges as a Platform for Precision Therapeutics.	Elnaggar et al. (2024), 38,302,049, Paunovska et al. (2022), 34,983,972, and Springer and Dowdy (2018), 29,792,572

*Note:* This table outlines the discoveries and milestones discussed in the Figure 2 timeline.

## 3 LncRNAs: Definition, Biogenesis, Structure and Functions

### 3.1 Transcriptional Origin, Classification, and Expression of LncRNAs

Non‐coding RNAs constitute a substantially larger proportion of the human transcriptome than coding RNAs (Clark et al. 2015). Ponting and Haerty estimated that only about 1.2% of the human transcriptome encodes proteins, meaning that approximately 98% consists of non‐protein‐coding transcripts (Ponting and Haerty 2022). LncRNAs are a broad class of RNA biomolecules confirmed to be transcribed in many plants, including Arabidopsis, corn, wheat, and rice, fungi, protozoa, and many, if not all, animal species (Akpinar et al. 2023; Arunkumar 2024; Lv et al. 2019). The term “lncRNA” originated in the early 2000s, following numerous publications that reported the prevalence of long noncoding transcripts in eukaryotic genomes.

**FIGURE 2:**
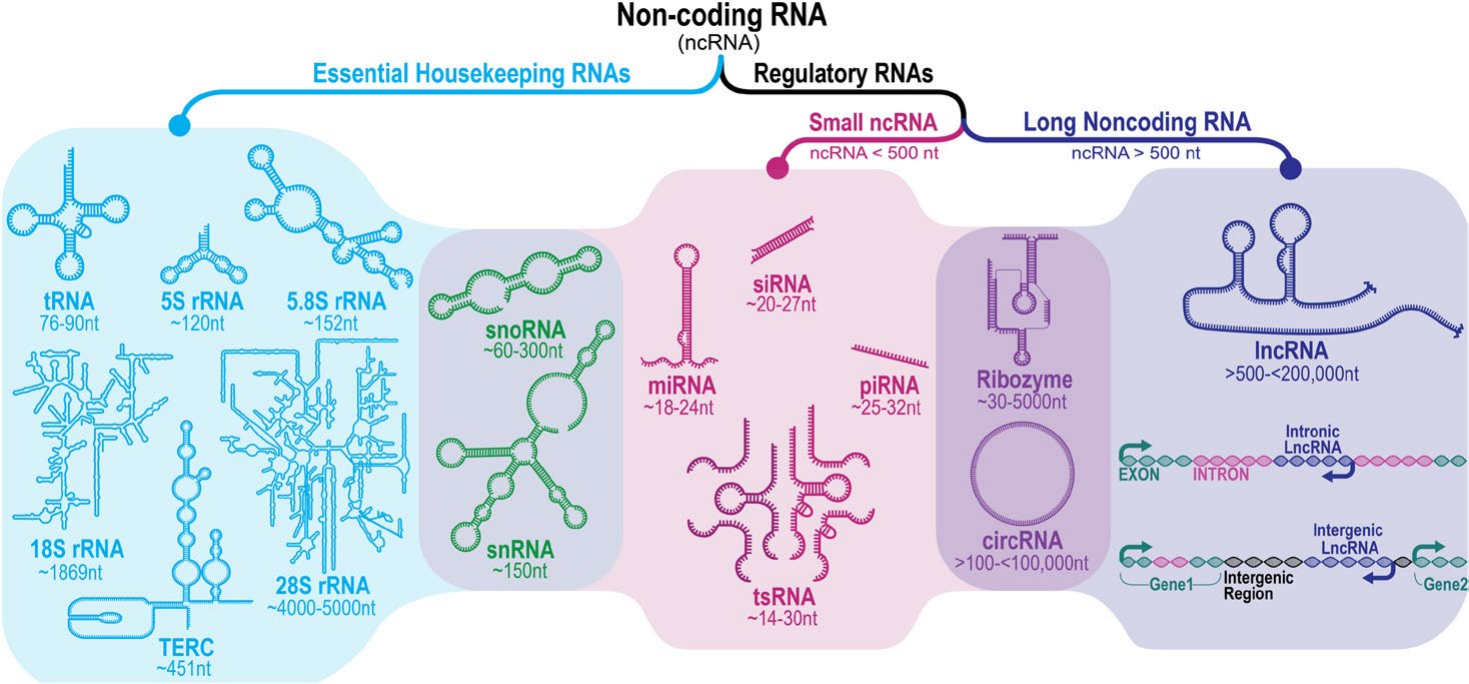
General Classification of Noncoding RNA Molecules. This schematic illustrates the major categories of noncoding RNA (ncRNA) based on size, function, and biogenesis. RNA species are broadly divided into essential housekeeping RNAs (light blue), small ncRNAs (ncRNAs <500 nt) (pink), and long noncoding RNAs (lncRNAs >500 nt) (dark blue). Example subtypes within each category are represented by name, approximate length, and representative structures. Abbreviations: circRNA, circular RNA; lncRNA, long non‐coding RNA; miRNA, microRNA; piRNA, PIWI‐interacting RNA; rRNA, ribosomal RNA; siRNA, small interfering RNA; snoRNA, small nucleolar RNA; snRNA, small nuclear RNA; TERC, Telomerase RNA Component; tRNA, transfer RNA; tsRNA, tRNA‐derived small RNA. Structures referenced from: (Amin et al. 2019; Chao et al. 2022; Du et al. 2023; Kwok et al. 2021; Mattick and Amaral 2022).

LncRNAs are broadly defined as RNA molecules longer than 200 nt that lack open reading frames capable of coding for proteins. This definition of lncRNAs was shaped by several practical constraints: distinguishing them from small RNAs, recognizing their unique regulatory roles, and establishing standards to facilitate detection (Cech and Steitz 2014). This definition set a standard that distinguished them from other RNA classes and provided a conceptual foundation for this emerging field of study. While lncRNAs are typically defined as greater than 200 nt, this limit was established somewhat arbitrarily, and a 500 nt cutoff is sometimes used, as it excludes some of the other categories of ncRNAs that would otherwise be included in the lncRNA category. The categorization of major ncRNA species based on general structural and functional features can be found in Figure 2.

In today's literature, the term lncRNA is used to describe an increasingly diverse set of ncRNAs that engage in a wide range of structural and functional roles. We now know that lncRNAs serve a wide range of functions and biological roles across an ever‐growing number of organisms. Specific terminology to describe separate or sub‐classes of lncRNA is common, but in many cases, it is not universal (Mattick et al. 2023). A simple search for “types of lncRNA” returns a host of different strategies by which to stratify lncRNAs: RNA polymerase, genomic location, function, subcellular localization, and others.

Our discussion of lncRNAs will focus on those transcribed by RNA Polymerase II (RNA pol II), categorize their common features and functions, and describe their implications across a variety of contexts.

#### 3.1.1 Biological Contexts

Although lncRNAs can be defined broadly enough to include ribosomal RNA rRNA and tRNA, these molecules are not considered here, as they carry out highly conserved roles and are transcribed by dedicated RNA polymerases distinct from RNA polymerase II. Specifically, ribosomal RNAs are transcribed by RNA polymerase I and transfer RNAs by RNA polymerase III, reflecting their distinct biogenesis, promoter elements, processing pathways and specialized transcriptional and functional constraints.

The number of lncRNAs in the human genome remains a subject of debate, with recent estimates ranging from approximately 16,000 annotated transcripts to over 100,000 putative transcripts. The wide range in estimates is due to experimental considerations regarding the inclusion or exclusion of unannotated transcripts and transcripts expressed in low abundance. Regardless of their exact number, ncRNAs are now accepted to be more abundant in the human transcriptome than coding RNAs (Statello et al. 2021). Although many lncRNAs are expressed in low abundance, expression level alone is a poor predictor of an lncRNA's functional relevance. Unlike protein‐coding genes, whose activity often correlates with transcript abundance, lncRNAs frequently act through highly localized mechanisms. The functions and interactions of lncRNAs can be governed by tissue‐ or cell‐type specificity, subcellular localization, or temporal regulation rather than by bulk expression levels (Cech and Steitz 2014; Statello et al. 2021). Numerous lncRNAs exhibit cell‐type specificity, developmental silencing, or act as response elements to environmental stimuli and stress response signaling pathways, suggesting that low steady‐state levels in bulk transcriptomic datasets obfuscate their critical regulatory roles in many biological contexts (Guttman et al. 2009; Mattick 2018; Ponting et al. 2009).

The genomic location of lncRNAs is generally described in reference to a nearby protein‐coding gene because of the intertwined nature of coding and noncoding transcription. Transcription of lncRNAs can occur from many genomic loci. LncRNAs can be intronic, exonic, intergenic (lincRNA), or overlap portions of another genomic locus, for example, the 3′ UTR of a coding gene. Transcription can occur in the sense or antisense direction relative to a nearby coding gene. LncRNAs are considered bidirectional if they are transcribed near (within 1 kb) and opposite to a coding gene transcriptional start site (Guttman et al. 2009; Katayama et al. 2005; Mattick et al. 2023). Although a greater proportion of lncRNAs are localized to the nucleus, lncRNAs can be either primarily nuclear or cytoplasmic in their localization. Differences in lncRNA localization may be the result of low lncRNA splicing efficiency and interactions between lncRNAs, chromatin, and chromatin‐associated proteins (Basu et al. 2023; Statello et al. 2021).

### 3.2 Cytoplasmic Functions of Long Noncoding RNAs

Nuclear localization is a defining feature of many lncRNAs; however, numerous lncRNAs also have functions in the cytoplasm, where they participate in post‐transcriptional regulation, ribonucleoprotein assembly, and translational control. One example is TUG1 and H19, which associate directly with ribosomes and ribosome‐bound translation complexes. These lncRNAs were previously characterized primarily as nuclear lncRNAs but were found to be enriched in ribosomal fractions, suggesting additional roles in regulating translation and polysome dynamics (van Heesch et al. 2014).

Another well‐characterized example is NORAD, a highly abundant cytoplasmic lncRNA that binds and sequesters Pumilio proteins, regulators of mRNA stability and chromosome segregation during mitosis. Loss of NORAD leads to excessive repression of Pumilio targets, disrupting cell‐cycle control and genomic stability (Tichon et al. 2016). Other cytoplasmic lncRNAs function as scaffolds, organizing signaling and regulatory complexes. LincRNA‐p21 interacts with RNA‐binding proteins and translational repressors to recruit RISC and modulate β‐catenin signaling, linking lncRNA activity and post‐transcriptional control of oncogenic pathways (Yoon et al. 2012). Similarly, the lncRNA HULC is enriched in the cytoplasm of hepatocellular carcinoma cells and regulates lipid metabolism and tumor‐associated signaling by acting as a sponge for multiple miRNAs (Wang et al. 2010). These studies show that lncRNAs extend regulatory control beyond the nucleus, influencing mRNA stability, translation, and signaling pathways central to cellular homeostasis and disease phenotype.

Many lncRNAs exhibit mRNA‐like processing features, including an N7‐methylguanosine (m7G) cap. The 3′ ends of many lncRNAs are heterogeneous or poorly defined, with only a subset containing functional polyadenylation sites and undergoing polyadenylation, a feature that influences transcript stability and degradation by the RNA exosome and other RNA surveillance pathways (Mattick et al. 2023; Mukherjee et al. 2023; Nojima and Proudfoot 2022). LncRNAs typically lack well‐conserved promoter regions with fewer transcription factor binding sites, leading to decreased transcription efficiency compared to mRNAs. LncRNAs are sometimes transcribed from regions with high nucleosome density, which can lead to decreased expression. Several factors contribute to the decreased stability of lncRNA transcripts, including weaker polyadenylation signals that cause premature transcriptional termination and inefficient splicing due to intron retention or non‐canonical splice sites. Finally, unlike mRNAs, many lncRNAs function in the nucleus, regulating local gene expression through chromatin remodeling, transcription factor regulation, or other mechanisms (Basu et al. 2023; Engreitz et al. 2016; Ulitsky and Bartel 2013).

### 3.3 LncRNAs Exhibit Broad Mechanisms of Gene Regulation

The functions of lncRNA span all facets of cellular and organismal physiology, including development, metabolic regulation, apoptotic control, cell cycle regulation, DNA damage response, tumor suppression, immune response, inflammation, and other homeostatic functions (Brannan et al. 1990; Desind et al. 2022; Kino et al. 2010; Liu et al. 2021; Santolucito et al. 2010; Wilusz et al. 2009; Zhou et al. 2012). LncRNAs act through several canonical mechanisms to impact gene expression. Some lncRNA transcripts are spliced to produce one or more miRNAs. For example, the lncRNA PVT1 encodes 5 separate miRNAs involved in oncogenesis and several hallmarks of cancer (Wu et al. 2023). LncRNAs also regulate miRNA availability through a process called sponging. This topic will be discussed in the MiRNA Biogenesis, Functions & Interactions With LncRNA section.

**FIGURE 3:**
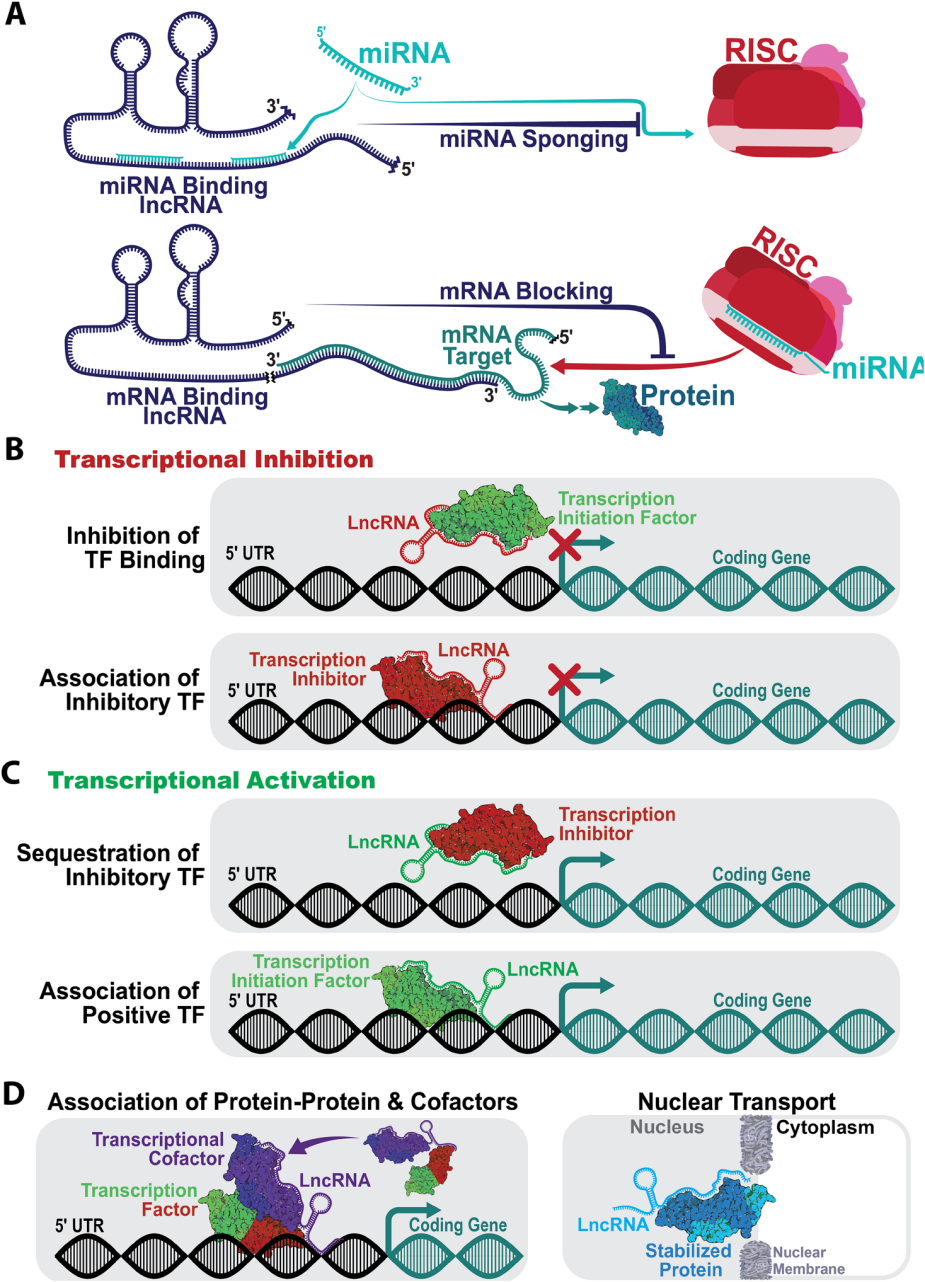
Mechanistic Diversity of LncRNAs in Post‐transcriptional and Transcriptional Regulation. This figure shows the diverse functions of lncRNAs in transcriptional and post‐transcriptional regulation. (A) Some lncRNAs act as “sponges” for miRNAs, physically binding and sequestering them away from their target mRNAs, preventing miRNA integration into the RNA‐induced silencing complex (RISC) complex and miRNA‐mediated mRNA degradation or translational inhibition. lncRNAs can also bind directly to mRNAs to form stable lncRNA‐mRNA complexes, thereby impacting ribosome access, splicing, miRNA binding, and mRNA stability, and thus modulating translation. (B) LncRNAs can negatively regulate gene expression by obstructing activating transcription factors, recruiting inhibitory transcription factors, or forming repressive complexes at target gene promoters and enhancers. (C) Conversely, lncRNAs can enhance transcription by blocking repressive transcription factors or serve as scaffolds for activating transcription factor complexes. (D) LncRNAs can act as scaffolds or molecular platforms that simultaneously interact with multiple proteins, transcription factors, cofactors for RNA polymerase II, chromatin‐modifying cofactors or other regulatory factors, to fine‐tune regulation of complex signaling networks. In the cytoplasm, lncRNAs may bind to and stabilize specific proteins, influencing their localization, function, or turnover, ultimately affecting downstream signal transduction and gene expression programs.

LncRNAs can regulate transcription by mediating DNA methylation, as seen with LINC00472, which suppresses tumorigenesis and metastasis in triple‐negative breast cancer (Shao et al. 2021). They also interact with transcription factors, either promoting transcription factor binding, such as the role of Meg3 in enhancing p53 signaling and endothelial function (Shihabudeen Haider Ali et al. 2019), or blocking transcription factor binding, for example, the lncRNA GAS5, which inhibits c‐Myc mRNA expression through interaction with the initiation factor 4E to promote tumor suppressive effects (Zhou and Chen 2020). Additionally, lncRNAs can modify chromatin or act as scaffolds, like HOTAIR, which coordinates histone modification complexes to silence gene expression (Rinn et al. 2007). These are just some of the many ways lncRNAs contribute to homeostatic maintenance. The major lncRNA mechanisms of gene regulation are summarized in Figure 3.

### 3.4 LncRNAs: The Structure–Function Relationship

lncRNAs influence transcription and translation through diverse mechanisms, including chromatin remodeling, mRNA stabilization, and numerous RNA and protein interactions, often in a cell‐type‐specific manner. While the nucleotide sequence is important for lncRNA underlying properties and stability, 3D conformation, including secondary or tertiary structure, is a defining determinant of their regulatory interactions and biological effects (Chillon and Marcia 2020; Desind et al. 2023; Mattick et al. 2023). Despite this, relatively few lncRNA structures have been resolved to date, underscoring a gap in our knowledge and understanding of the lncRNA structure–function relationship (Sanbonmatsu 2022).

Early efforts to determine RNA structure relied on enzymatic digestion by RNase or chemical probing with DMS. A significant advance came with the development of selective 2′‐hydroxyl acylation analyzed by primer extension (SHAPE) around 2005. SHAPE uses electrophilic reagents such as 1‐Methyl‐7‐nitroisatoic anhydride (1 M7), 5‐nitroisatoic anhydride (5NIA), or 2‐aminopyridine‐3‐carboxylic acid imidazolide (2A3) that react preferentially with flexible or unbound nucleotides in the RNA backbone, producing stops or mutations during reverse transcription that indicate structurally flexible regions (Wilkinson et al. 2006). More recently, SHAPE chemistry has been combined with high‐throughput sequencing strategies, such as mutational profiling (MaP), to form the SHAPE‐MaP approach.

SHAPE‐MaP utilizes robust high‐fidelity reverse transcriptase with strong read‐through capability. At SHAPE‐modified nucleotides, the reverse transcriptase incorporates random mutations, which are quantified by deep sequencing (Wilkinson et al. 2006). The SHAPE‐MaP strategy has been used to resolve the secondary structures of large lncRNAs and even whole transcriptomes with accuracy comparable to traditional analyses of small model RNAs (Marinus et al. 2021; Smola et al. 2016; Wilkinson et al. 2006). Strategies such as SHAPE‐MaP, combined with computational tools, have greatly expanded our ability to visualize and quantify lncRNA structures, enabling structure–function characterization in more complex RNAs. Several well‐known lncRNAs have been structurally interrogated using SHAPE or similar probing strategies, such as MALAT1, HOTAIR, and NEAT1, which will be discussed in more detail below.

Experiments using SHAPE‐MaP and other methods of structural analysis have shown that lncRNAs often fold into modular architectures composed of structured domains connected by flexible linkers. They also exhibit long‐range interactions, allowing for higher‐order structural organization. These features enable lncRNAs to function as molecular scaffolds, protein‐binding platforms, or regulatory hubs whose activity depends on spatial organization rather than primary sequence alone.

### 3.5 Structural Probing & Functional Importance of Well‐Known LncRNAs

To illustrate how these structural principles manifest in biologically relevant contexts, several lncRNAs have been examined in detail using integrated structural and functional approaches. MALAT1, HOTAIR, and NEAT1 represent well‐characterized examples in this regard, each with defined structural domains mechanistically linked to protein binding, nuclear organization, and transcriptional control. These lncRNAs serve as representative models demonstrating how RNA folding principles underlie lncRNA function across diverse regulatory contexts.

MALAT1 (Metastasis‐Associated Lung Adenocarcinoma Transcript 1) is an approximately 8 Kb nuclear lncRNA first identified in lung cancer. Structural studies have revealed that the 3′ end of MALAT1 is not polyadenylated but instead forms a highly conserved 76‐nt triple helix that protects the transcript from exonucleolytic degradation (Monroy‐Eklund et al. 2023). SHAPE‐MaP profiling of full‐length MALAT1 using 5NIA (in vivo and ex vivo) further showed that MALAT1 adopts well‐defined secondary structural domains that are conserved across different cell types. MALAT1's structure regions are also conserved across primate species despite nucleotide sequence divergence. This conserved architecture is thought to facilitate MALAT1's role in regulating splicing and metastasis‐related signaling networks, including MAPK/ERK, TGF‐β/SMAD and PI3K/AKT, STAT3 and NF‐κB by providing structural platforms for protein and RNA interactions (Feichtenschlager et al. 2023; Hou et al. 2023; Monroy‐Eklund et al. 2023; Zhang et al. 2020). Consistent with a structure‐dependent role, loss of MALAT1 in the MMTV‐PyMT mouse mammary carcinoma model slowed tumor growth, reduced metastasis, and promoted differentiation into cystic tumors. MALAT1 depletion also altered gene expression and splicing of pathways controlling differentiation and cell migration, indicating that MALAT1's conserved RNA architecture is required to maintain oncogenic cell states (Arun et al. 2016).

Another well‐known lncRNA, HOTAIR, has established roles in cancer metastasis and gene silencing and was structurally resolved using SHAPE. SHAPE analysis using 1M7 of the 2148 nt HOTAIR transcript identified a detailed and modular (discrete self‐contained domains) secondary structure. SHAPE and DMS probing combined with phylogenetic analysis revealed that HOTAIR folds into four independent conserved domains, two of which corresponded to its known protein‐binding regions (the 5′ domain binding Polycomb repressive complex 2 (PRC2) and the 3′ domain binding LSD1/CoREST complex) (Kim et al. 2020). Notably, the structured hairpins surrounding these protein‐binding motifs are evolutionarily conserved between human and mouse, suggesting that HOTAIR's secondary structure is critical for recruiting chromatin‐modifying complexes (Somarowthu et al. 2015). This structural map of HOTAIR has provided an outline for targeted mutagenesis studies, which confirmed that specific hairpin structures were required for HOTAIR's gene‐silencing function. The modular structural organization of HOTAIR functions as an interface between chromatin and repressive epigenetic machinery. HOTAIR is upregulated in primary breast tumors and metastases, where its expression predicts metastatic progression and poor outcome. Enforced HOTAIR expression drives genome‐wide retargeting of Polycomb Repressive Complex 2 (PRC2), altering H3K27 methylation and gene expression to promote invasiveness and metastasis, whereas HOTAIR depletion suppresses these phenotypes in a PRC2‐dependent manner (Gupta et al. 2010).

NEAT1 is a nuclear lncRNA (with two major isoforms of 3.7 Kb and 23 Kb) that is essential for the assembly of paraspeckles (subnuclear ribonucleoprotein granules) and is highly expressed in multiple epithelial tissues. SHAPE structural analysis of the NEAT1 secondary structure using 1 M7 indicates that the short isoform folds into at least four modular domains (Lin et al. 2018). While the overall secondary structure is only partly conserved between human and mouse, NEAT1's function and role in paraspeckle formation remain similar (Lin et al. 2018). This conservation of structure suggests that NEAT1's ability to act as a nuclear scaffold does not result from sequence and local base‐pairing conservation and instead relies on higher‐order interactions, such as long‐range RNA–RNA interactions between its 5′ and 3′ ends. These structural features of NEAT1 have been predicted in silico and validated in vitro, supporting a model in which NEAT1 ends pair to bring its distal regions into proximity (Lin et al. 2018; Taiana et al. 2020). Structural modeling of NEAT1 using SHAPE probing has shown that lncRNAs that function as structural scaffolds can rely on the conservation of discrete domains that govern RNA architecture, despite a lack of global sequence conservation.

Here, we discussed how lncRNA function is tightly coupled to RNA structure and higher‐order organization. Advances in chemical probing approaches such as SHAPE‐MaP have enabled transcriptome‐scale interrogation of lncRNA secondary structure, revealing conserved, modular architectures that underlie regulatory activity. Structural studies of MALAT1, HOTAIR, and NEAT1 demonstrate how RNA folding, rather than primary sequence alone, governs transcript stability, protein interactions, and nuclear organization. Collectively, these findings establish RNA structure as a central determinant of lncRNA mechanisms in gene regulation and disease.

## 4 MiRNA Biogenesis, Functions and Interactions With LncRNA

### 4.1 Biogenesis of MicroRNAs, Their Processing & Regulatory Functions

In 1993, Dr. Victor Ambros' research group discovered the first miRNA in *C. elegans* (Lee et al. 1993). They identified a small RNA named lin‐4, which negatively regulates the lin‐14 gene by binding with a complementary sequence in the lin‐14 3′ UTR (described earlier in this review). This conserved class of ncRNAs was named “miRNAs,” due to their short length (typically 18–22 nt). There are now over 2600 miRNAs cataloged in the miRbase database, which regulate gene expression in all human tissues and contribute to hundreds of documented diseases and disorders (Kozomara et al. 2019). Advancements in structural biology techniques, combined with advances in sequencing technology, have accelerated our understanding of the molecular mechanisms underlying miRNA biogenesis and their roles in cancer and many other diseases.

**FIGURE 4:**
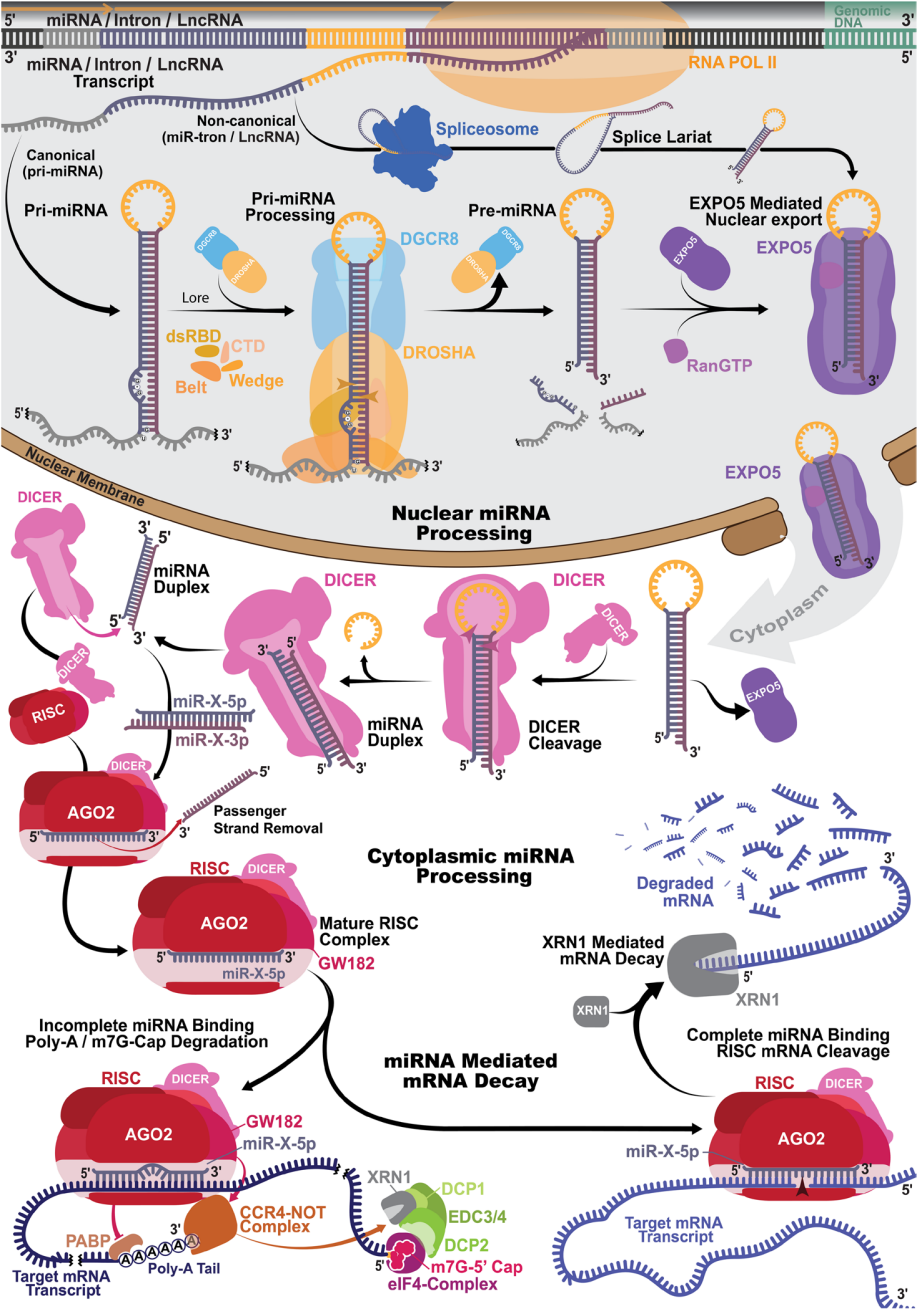
Canonical and Non‐canonical miRNA‐Mediated mRNA Decay Pathway. This figure illustrates the canonical and select non‐canonical pathways by which miRNAs are transcribed, processed, exported, and ultimately loaded into effector complexes that regulate target mRNAs. At the top, nascent primary miRNA (pri‐miRNA) transcripts are generated by RNA polymerase II from dedicated miRNA genes, intronic regions or lncRNA loci. Non‐canonical miRNA biogenesis can arise via splicing events from introns or lncRNAs. In the nucleus, the Microprocessor complex, consisting of DROSHA (yellow‐orange) and DGCR8 (blue), recognizes and cleaves the pri‐miRNA to yield a 60‐120 nt precursor miRNA (pre‐miRNA) hairpin. Exportin‐5 (EXP5) in complex with RanGTP binds the pre‐miRNA and is transported to the cytoplasm. Once in the cytoplasm, the ribonuclease III enzyme DICER (pink) trims the pre‐miRNA into an ~22 nt double‐stranded miRNA duplex. One strand (the “guide” strand) is selectively loaded into Argonaute 2 (AGO2, red) to form the RNA‐induced silencing complex (RISC). The other strand (the “passenger” strand) is typically discarded. The guide miRNA directs RISC to complementary sequences in target mRNAs, leading to post‐transcriptional repression or mRNA degradation. Incomplete binding of the miRNA guide strand results in mRNA decay through deadenylation mediated by GW182 and the CCR4‐NOT complex and decapping by enzymes DCP1, DCP2, EDC3/4, exonuclease1 (XRN1), and other associated factors. Complete binding of the miRNA guide strand to the miRNA results in mRNA cleavage by AGO2 and mRNA degradation by XRN1 and other exonucleases. Mechanisms referenced from: (Nguyen et al. 2015; Shang et al. 2023; Wurm and Sprangers 2019; Yamazawa et al. 2018).

Although miRNAs are present in all eukaryotic lineages, miRNA processing differs throughout the major evolutionary branches (Axtell et al. 2011). In humans, the canonical biogenesis of miRNAs is a complex, multi‐step process that originates in the nucleus and involves the transcription of long primary pri‐miRNA transcripts by RNA Polymerase II (Figure 4). Transcription can occur from multiple locations within the genome. Canonical miRNAs exist as independent genes with their own promoters and transcription factors. These transcripts, called pri‐miRNAs, are capped, polyadenylated, and folded into a hairpin structure. Non‐canonical miRNAs exist as part of the primary transcript of another gene. Nearly half of miRNAs are miRtrons; miRNAs transcribed from the intronic regions of protein‐coding genes (Alles et al. 2019; Gulyaeva and Kushlinskiy 2016). lncRNAs are another source of primary transcripts for miRNA biogenesis. Researchers in the Cullen lab discovered a 23‐nucleotide miRNA derived from the first known imprinted ncRNA, H19, in both humans and mice. They demonstrated that H19 serves as a primary miRNA precursor for miR‐675, suggesting its role in post‐transcriptional regulation of developmentally regulated gene expression (Cai and Cullen 2007). Mentioned earlier, the lncRNA PVT1 also encodes several miRNAs (miR‐1208, miR‐1207‐3p, miR‐1207‐5p, miR‐1206, miR‐1205, and miR‐1204), which have both oncogenic and tumor‐suppressive roles in multiple cancers influenced by feedback from PVT1 (Wu et al. 2023). While there are many lncRNAs known to act as miRNA precursors, exactly how these transcripts are processed is not entirely understood, partly due to the complex tissue and cell‐type‐specific functions of both lncRNAs and miRNAs.

Canonical pri‐miRNAs are processed within the nucleus by the microprocessor complex. The key components of this complex, the RNase III enzyme Drosha and its binding partner DGCR8, work in concert with several other components (CTD, Belt, dsRBDs, Wedge) to recognize and cleave the pri‐miRNA near the base of its hairpin stem (Shang et al. 2023). This process produces a truncated precursor miRNA (pre‐miRNA) intermediate. This pre‐miRNA is approximately 60–70 nucleotides long and has a 2‐nt 3′ overhang. Alternative processing of miRtrons and lncRNA‐derived miRNAs by the spliceosome typically bypasses cleavage by Drosha, becoming pre‐miRNAs through splicing and lariat debranching (O'Brien et al. 2018).

After nuclear processing, pre‐miRNAs are shuttled into the cytoplasm via Exportin‐5 (EXPO5) in a RanGTP‐dependent manner. Once in the cytoplasm, Dicer, another RNase III enzyme, trims the pre‐miRNA loop and releases the duplexed passenger strand and complementary 18–22 nt mature miRNA guide strand. The duplex is incorporated into an Argonaute 2 (AGO2) protein complex, and the less stable passenger strand is preferentially removed, yielding a mature RNA‐induced silencing complex (RISC). The mature RISC complex is made up of a large number of proteins, including Dicer and GW182, which can interact with cytoplasmic poly(A)‐binding protein (PABP), the CCR4‐NOT deadenylase complexes, and downstream DCP1/2 decapping complexes (Behm‐Ansmant et al. 2006). The mature miRNA guides the RISC complex and seeks out complementary sites within the 3′ UTRs of target mRNAs or lncRNAs. Less commonly, miRNAs can also bind to other regions of transcripts, including the 5′ UTR and coding regions, to regulate gene expression (O'Brien et al. 2018). Once a target transcript is acquired, RISC silences gene expression through several defined interlinked mechanisms.

Complete binding of the miRNA guide strand to the target transcript results in cleavage of the target by AGO2 and subsequent degradation by Exoribonuclease 1 (XRN1), or, in the context of mRNA, blocking of translation. More commonly, the 3′ and 5′ ends of the miRNA guide strand partially bind to the target transcript. Partial binding of the miRNA guide results in activation of downstream pathways through GW182 and the CCR4‐NOT deadenylation complex, which facilitates poly(A) tail removal, activation of decapping complexes DCP1/2, mRNA destabilization, and subsequent exonucleolytic degradation of the target (Behm‐Ansmant et al. 2006; Valencia‐Sanchez et al. 2006). The degree of sequence complementarity between the miRNA guide and its target transcript influences the strength and stability of the RISC‐mediated response, enabling high selectivity and control over transcript repression, degradation, and downstream translation (Shang et al. 2023). In some cases, target binding can also lead to miRNA decay. During target‐directed microRNA degradation (TDMD), transcripts with extensive complementarity to the miRNA‐loaded AGO complex destabilize the AGO‐bound miRNA through tailing, trimming, and ZSWIM8‐associated turnover, mechanisms of selective miRNA decay (Ameres et al. 2010; Shi et al. 2020). MiRNA biogenesis and function are summarized in Figure 4.

MiRNAs play critical roles across a wide range of diseases and disorders, but many are aberrantly expressed and functionally significant in cancer. Deletion of the miR‐15a/16–1 cluster within the 13q14 locus drives B cell‐autonomous lymphoproliferative disorders in mice that recapitulate human chronic lymphocytic leukemia, establishing this cluster as a tumor suppressor through control of cell‐cycle gene expression (Klein et al. 2010). In contrast, oncogenic miRNAs such as miR‐21 are broadly overexpressed across solid tumors, where they repress tumor suppressors including RB1 and TGFBR2 (Volinia et al. 2006). Conditional in vivo overexpression of miR‐21 is sufficient to induce malignancy, whereas miR‐21 inactivation results in rapid tumor regression, highlighting its central role in sustaining oncogenic gene‐expression programs (Medina et al. 2010).

MiRNA regulation also plays a central role in regulating inflammatory signaling (Desind et al. 2023; Iacona et al. 2018; Monteleone and Lutz 2021; Tu and Hu 2021). In inflammatory and therapeutic contexts, miR‐708‐5p acts as a tumor‐suppressive regulator of TNFα/IL‐1β and COX‐2/PGE_2_ signaling by targeting key nodes, including IKKβ, COX‐2, and mPGES‐1, thereby reducing NF‐κB activation, limiting prostaglandin production, and restoring chemosensitivity in lung cancer models (Monteleone and Lutz 2021). Collectively, these examples illustrate how dysregulated miRNAs can function as dominant drivers of oncogenesis, inflammation, and therapeutic response.

### 4.2 LncRNA and MiRNA Interactions

**FIGURE 5:**
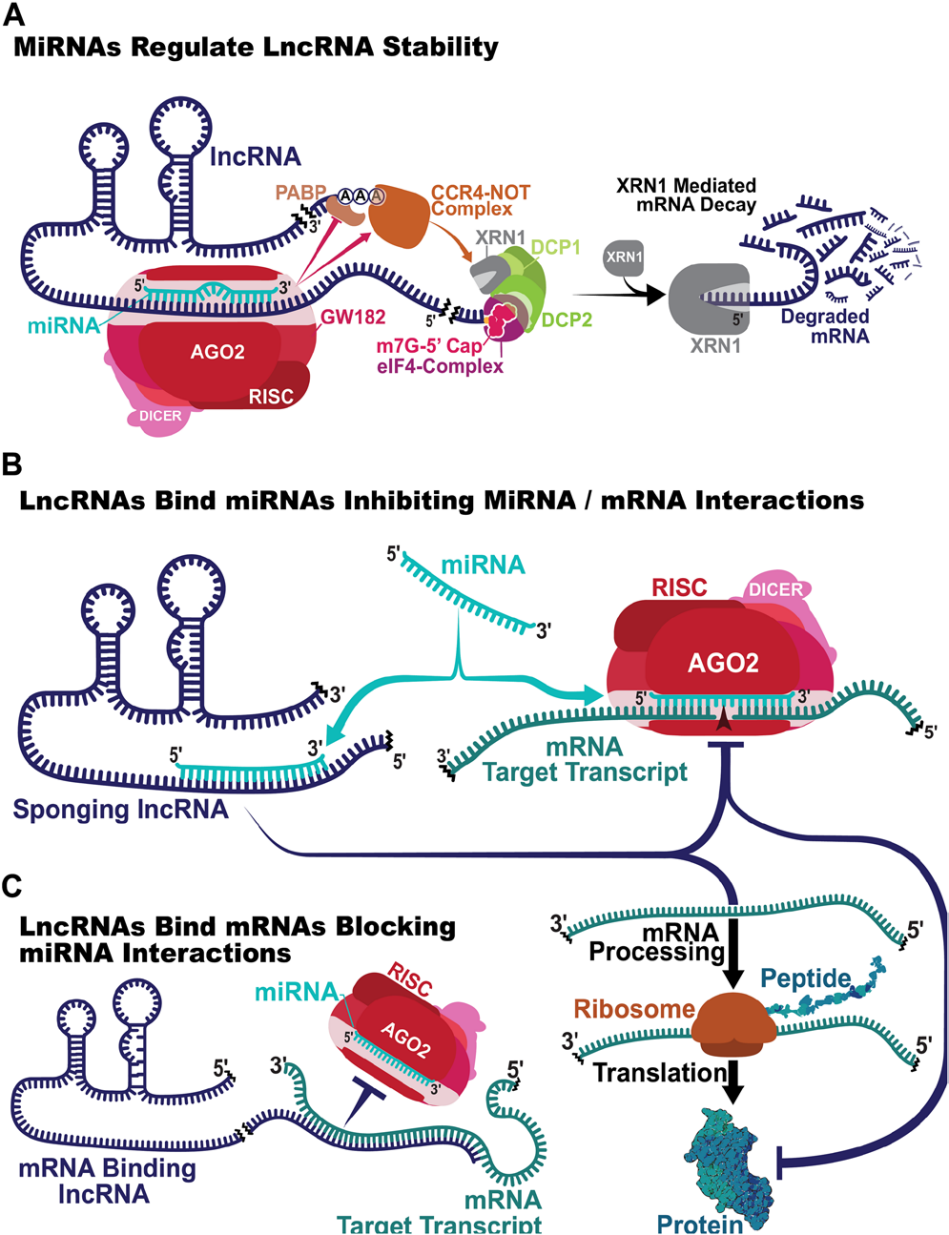
LncRNA‐MiRNA Interactions Regulate Gene Expression. (A) Mature miRNA is loaded into AGO2, forming the RNA‐induced silencing complex (RISC). Base‐pairing between the miRNA and target lncRNA results in the recruitment of the CCR4‐NOT deadenylation complex, lncRNA decapping by DCP1 and DCP2, and exonucleolytic degradation by XRN1 (gray). This causes transcript destabilization and degradation. (B) LncRNAs can “sponge” miRNAs by presenting multiple decoy miRNA‐binding sites, sequestering miRNAs away from their mRNA targets. Sponging effectively prevents miRNA complexing with AGO2, decreasing miRNA‐mRNA interactions and increasing levels of target mRNA and ribosome‐mediated translation. (C) LncRNAs can bind directly to mRNAs, stabilizing the transcript by blocking access to the RISC complex.

The interactions between lncRNAs and miRNAs form complex, tissue‐ and cell‐type‐specific regulatory networks that impact many biological processes in both healthy and disease states (Figure 5). MiRNAs can regulate lncRNA transcripts through the RISC pathway, described in the previous section. Similar to mRNA transcript regulation, RISC can associate with lncRNAs, leading to cleavage or destabilization of the lncRNA target transcript. For example, in renal cell carcinoma cells, miR‐141 binds directly to HOTAIR, a well‐studied oncogenic lncRNA, leading to decreased HOTAIR transcript levels and attenuated cancer cell proliferation and invasion (Chiyomaru et al. 2014).

Conversely, lncRNAs can bind to and sequester miRNAs, thereby disrupting the interactions between these miRNAs and their target mRNA transcripts. This process is commonly referred to as miRNA sponging. These lncRNA sponges effectively reduce the pool of freely available miRNAs that would otherwise target protein‐coding transcripts. Sponging can occur in the local vicinity of active gene transcription, but lncRNAs typically diffuse throughout the cytoplasm to broadly regulate miRNA expression (Feng et al. 2022). MiRNA binding sites present on lncRNAs are specific for a particular miRNA or family of miRNAs. The physical accessibility and degree of complementarity between the binding site and a particular miRNA determine an lncRNA's ability to protect an mRNA target from repression and enhance translation (Chan and Tay 2018; Tay et al. 2014; Thomson and Dinger 2016). LncRNAs can also bind directly to mRNA transcripts, acting as a shield by blocking miRNA binding. One example of this is the interaction between the paxillin antisense 1‐L (PAX‐AS1‐L) lncRNA and paxillin (PXN), in which PAX‐AS1‐L stabilizes PXN, thereby increasing the risk of hepatocellular carcinoma (Yuan et al. 2017). Figure 5 summarizes the types of lncRNA/miRNA interactions discussed above.

## 5 Developments in RNA Therapeutics

### 5.1 Clinical Translation of RNA Therapeutics and Liver‐Directed Delivery

Our understanding of ncRNAs has rapidly evolved, leading to the development of therapeutic strategies that utilize their structural, specific binding, and mechanistic functions to target mRNA, proteins, and other RNA species. NcRNA‐based therapeutics utilizing RNAi pathways have repeatedly demonstrated success across multiple treatment strategies, including antisense oligonucleotides (ASOs) and small interfering RNAs (siRNAs). While the recent mRNA vaccines encoding the spike protein of the SARS‐CoV‐2 virus are not ncRNA‐based, they have drawn significant attention to the importance of RNA‐based therapeutics (Egli and Manoharan 2023; Hou et al. 2021; Paunovska et al. 2022). Multiple successful clinical trials and regulatory approvals over the past decade have demonstrated that RNA drugs can achieve prolonged, sustained treatment effects in humans (Adams et al. 2018; Finkel et al. 2017; Paunovska et al. 2022; Polack et al. 2020; Syed 2025). Currently, the primary constraints on the development of clinically available therapeutics are treatment delivery, efficiency, tissue specificity, tolerability, and long‐term safety (Elnaggar et al. 2024; Paunovska et al. 2022).

The current, most established, and clinically successful RNA therapeutics rely on hepatic delivery, due to both favorable hepatic uptake and established regulatory precedent. Hepatocytes are easily accessible from systemic circulation and express high levels of endocytic receptors, notably the asialoglycoprotein receptor (ASGPR), allowing for efficient uptake of circulating ligand and efficient delivery of RNA therapeutics (Elnaggar et al. 2024; Lamb 2021; Paunovska et al. 2022; Springer and Dowdy 2018). FDA approvals of N‐acetylgalactosamine (GalNAc) ‐conjugated ASOs and siRNAs targeting hepatic genes such as TTR (transthyretin), APOC3 (Apolipoprotein C‐III), and SERPINC1 (Serpin Family C Member 1) illustrate that this delivery strategy enables sustained target suppression with a periodic but infrequent dosing schedule, supporting long‐term treatment of metabolic, hematologic, and rare genetic disorders (Hoy 2018; Lee 2025; Paunovska et al. 2022; Syed 2025). Conjugation of oligonucleotides to triantennary GalNAc (tri‐GalNAc) ligands enables highly specific uptake by hepatocytes through the ASGPR receptor, resulting in potent gene silencing following subcutaneous administration.

Advances in oligonucleotide design have improved nuclease resistance, pharmacokinetics, tissue distribution, and durability across both ASO and siRNA strategies. Incorporation of phosphorothioate linkages and 2′‐ribose modifications such as 2′‐O‐methyl and 2′‐O‐methoxyethyl enhances metabolic stability, improves cellular uptake, and reduces innate immune activation. ASOs containing a central DNA region flanked by chemically modified wings, known as gapmers, enable efficient RNase H‐mediated target RNA cleavage. By contrast, siRNA activity depends on strand selection rather than catalysis by recruited complexes. Engineered duplex asymmetry of siRNAs promotes selective guide‐strand loading into RISC, thereby increasing silencing specificity and potency (Crooke et al. 2020; Khvorova and Watts 2017; Springer and Dowdy 2018).

### 5.2 Expanding RNA Therapeutics Beyond the Liver

While hepatic delivery dominates current approvals, the major frontier for RNA therapeutics lies in achieving reliable and safe tissue‐specific targeting beyond the liver (Elnaggar et al. 2024). Delivery to the lung, central nervous system, immune compartments, and solid tumors remains limited by biodistribution, cellular uptake, endosomal escape, and dose‐limiting toxicities (Elnaggar et al. 2024; Gilleron et al. 2013; Paunovska et al. 2022). Tissue‐selective lipid nanoparticles represent one strategy to address this barrier, as subtle changes in nanoparticle composition can bias organ distribution and expand extrahepatic RNA delivery (Cheng et al. 2020; Hofstraat et al. 2025). Hofstraat et al. show that engineered apolipoprotein A1 nanoparticles can deliver ncRNA therapeutics (siRNA and ASOs) beyond the liver to bone‐marrow myeloid cells and their progenitors in vivo. They achieved functional knockdown of Lamp1 in bone marrow stem/progenitor and myeloid cells, and therapeutic silencing of Ccr2, reducing the recruitment of immunosuppressive monocytes/macrophages in a syngeneic tumor model after systemic administration (Hofstraat et al. 2025).

CRISPR systems are inherently ncRNA‐based, relying on sequence‐specific guide RNAs to target and direct precise genomic modification (Gillmore et al. 2021). In vivo delivery of guide RNAs and mRNA encoding genome‐editing enzymes via lipid nanoparticles enables permanent modification of disease‐associated genes after a single administration (Cheng et al. 2020; Gillmore et al. 2021). The MAGNITUDE‐2 trial (2023) is evaluating a one‐time CRISPR‐based therapy, nexiguran ziclumeran (NTLA‐2001), for transthyretin amyloid cardiomyopathy, while related base‐editing strategies (VERVE‐101 / VERVE‐102) target PCSK9 (proprotein convertase subtilisin/kexin type 9) to treat hypercholesterolemia and atherosclerotic cardiovascular disease, including heterozygous familial hypercholesterolemia and premature coronary artery disease. Lipid nanoparticle‐delivered CRISPR base editors introduce a single‐nucleotide disruption in PCSK9, permanently suppressing expression and lowering LDL cholesterol after one dose (Hooper et al. 2024; Tual‐Chalot and Stellos 2024). These programs underscore both the promise and clinical complexity of in vivo RNA‐guided gene editing and the need for long‐term safety monitoring.

**FIGURE 6:**
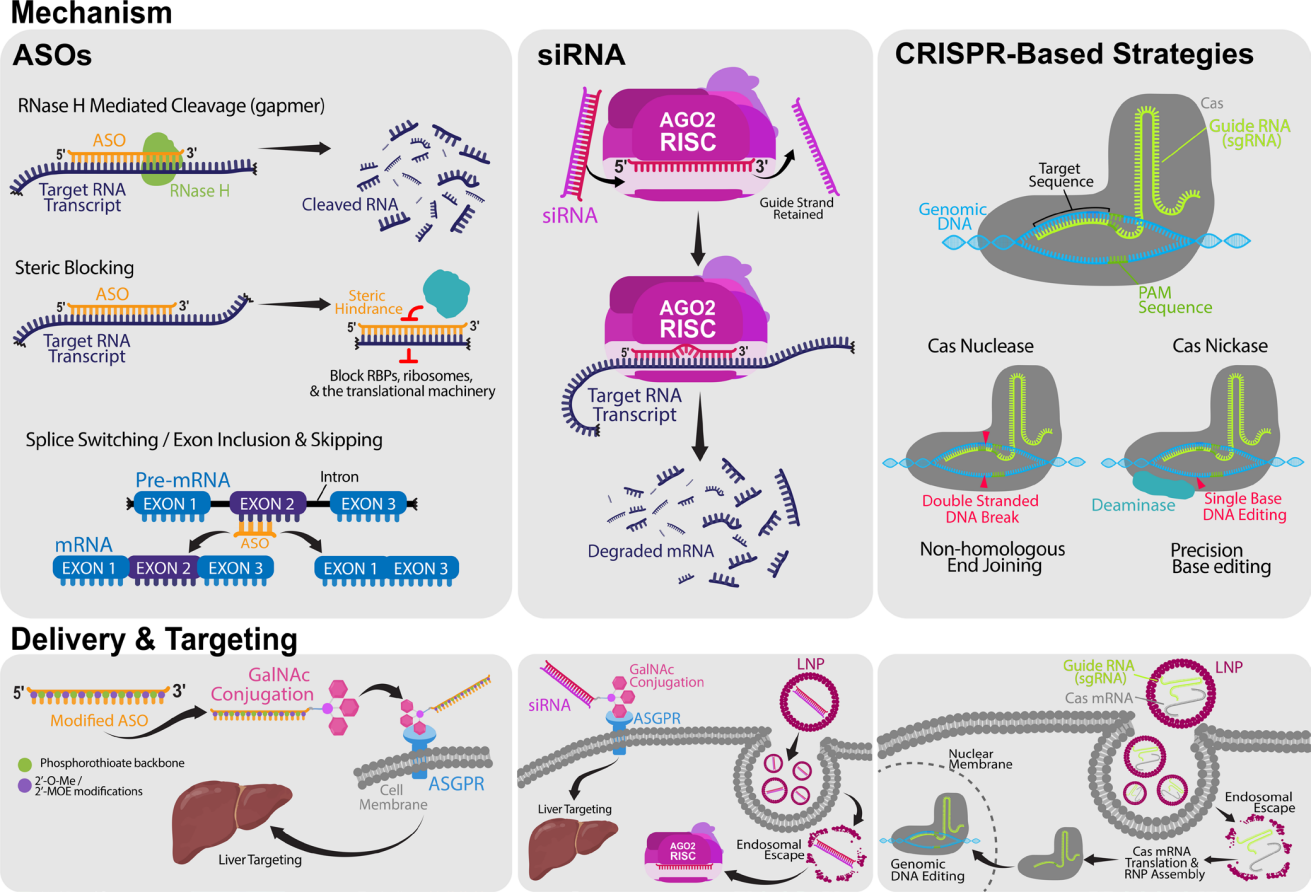
RNA Therapeutic Mechanisms: Delivery and Targeting. Schematic comparison of ASO, siRNA, and CRISPR‐based therapeutic strategies described in the text. ASOs bind target RNA transcripts and can induce RNase H‐mediated cleavage, steric blocking of RNA‐binding proteins and translational interactions or modulate pre‐mRNA splicing. Chemical modifications, including phosphorothioate backbones and 2′‐O‐Me substitutions, improve ASO stability and pharmacologic performance, while GalNAc conjugation supports ASGPR‐mediated hepatocyte targeting. siRNAs are loaded into AGO2/RISC, where the retained guide strand directs target mRNA cleavage and degradation. siRNA delivery is commonly supported by GalNAc‐mediated liver targeting or lipid nanoparticles, although endosomal escape remains a major limitation. CRISPR‐based approaches use guide RNAs to direct Cas nucleases or Cas nickase‐deaminase base editors to a specific genomic DNA target near a PAM sequence, enabling gene disruption or precise base editing. LNP delivery of guide RNA and Cas/editor mRNA supports in vivo genome editing, with liver delivery currently the most clinically advanced strategy. Abbreviations: 2′‐MOE, 2′‐O‐methoxyethyl; 2′‐O‐Me, 2′‐O‐methyl; AGO2, Argonaute 2; ASGPR, asialoglycoprotein receptor; ASO, antisense oligonucleotide; Cas, CRISPR‐associated protein; CRISPR, clustered regularly interspaced short palindromic repeats; GalNAc, N‐acetylgalactosamine; LNP, lipid nanoparticle; PAM, protospacer adjacent motif; RISC, RNA‐induced silencing complex; RNase H, ribonuclease H; sgRNA, single‐guide RNA; siRNA, small interfering RNA.

Despite substantial progress, several challenges remain central to the future of RNA therapeutics. Efficient endosomal escape remains a major bottleneck for most systemic delivery platforms, while achieving consistent extrahepatic targeting without immune activation or toxicity remains an unsolved problem across many tissues (Elnaggar et al. 2024; Gilleron et al. 2013; Paunovska et al. 2022). In addition, RNA‐delivered gene editing raises unique regulatory and safety considerations, as permanent genomic modifications necessitate careful risk–benefit evaluation and long‐term patient monitoring (Gillmore et al. 2021; *NCT06128629*, 2023). Some of the mechanisms and strategies discussed in this section are outlined in Figure 6. Even with these challenges, recent developments indicate the continued expansion of RNA therapeutics as a versatile drug class with the potential to reshape treatment standards for many conditions.

## 6 Conclusion

RNA research has progressed from early observations of abundant cytoplasmic ribonucleic acid to recognition of ncRNAs as central regulators of gene expression, cell fate, and disease. A central theme that emerges from this analysis of ncRNA across time and function is that regulatory control by defined ncRNA classes is distributed across distinct but overlapping mechanisms. MiRNAs provide a conserved layer of Argonaute‐dependent post‐transcriptional regulation, whereas lncRNAs regulate chromatin state, transcription, nuclear organization, and post‐transcriptional processes. Additional ncRNA classes, including snoRNAs, snRNAs, circRNAs, and eRNAs, further shape gene regulation by directing RNA processing and modification, coordinating splicing, organizing regulatory compartments, and coupling transcription to chromatin architecture. Across these classes, ncRNA function is highly context dependent, dictated by genomic origin, subcellular localization, and higher‐order RNA structure, with interactions between lncRNAs and miRNAs integrating transcriptional and post‐transcriptional control to amplify modest changes in RNA abundance into substantial phenotypic effects (Cech and Steitz 2014; Huntzinger and Izaurralde 2011; Kiss‐László et al. 1996; Statello et al. 2021; Tollervey 1987).

From a translational perspective, efficient and tissue‐specific delivery remains the primary constraint limiting the therapeutic application of ncRNAs. Clinically validated RNA modalities remain concentrated in platforms with predictable biodistribution, most notably GalNAc‐siRNA conjugates targeting hepatocytes and lipid nanoparticle systems used for select RNA therapeutics, including RNA‐guided gene editing and mRNA vaccines (Elnaggar et al. 2024; Paunovska et al. 2022; Springer and Dowdy 2018; Statello et al. 2021). Consequently, RNA therapeutic development is still largely guided by delivery feasibility rather than biological target relevance. Clinical testing, therefore, remains focused on tissues with established delivery strategies, even as emerging ncRNA‐directed strategies, including siRNA, antisense, and RNA‐guided genome editing, expand the therapeutic landscape. Future progress in miRNA‐ and lncRNA‐based therapeutics will depend on the development of programmable extrahepatic delivery platforms that enable reproducible targeting beyond the liver and limited immune compartments (Elnaggar et al. 2024; Hou et al. 2021).

NcRNAs function within coordinated regulatory networks shaped by RNA structure, protein interactions, genomic context, and subcellular localization, with regulatory activity encoded either in base‐specific interactions or higher‐order structural domains. MiRNAs provide a conserved layer of post‐transcriptional regulation through sequence‐directed targeting, whereas lncRNAs modulate chromatin organization, transcription, and RNA‐protein interactions with cell‐ and tissue‐specific effects. These properties underscore the need for continued mechanistic and structural investigation of miRNAs and lncRNAs to accurately model gene regulatory networks and define their roles in human biology and disease.

## Author Contributions

**Samira K. Bell:** conceptualization, writing – review and editing, writing – original draft, funding acquisition. **Carol S. Lutz:** conceptualization, writing – review and editing, project administration, supervision, resources, funding acquisition. **Samuel Z. Desind:** conceptualization, writing – original draft, writing – review and editing, visualization, project administration, investigation, funding acquisition.

## Funding

This work was supported by the Rutgers School of Graduate Studies and the New Jersey Commission on Cancer Research (NJCCR; COCR23PRF015, COCR26PRF019).

## Conflicts of Interest

The authors declare no conflicts of interest.

## Data Availability

Data sharing not applicable to this article as no datasets were generated or analysed during the current study.
